# Regional Admixture Mapping and Structured Association Testing: Conceptual Unification and an Extensible General Linear Model

**DOI:** 10.1371/journal.pgen.0020137

**Published:** 2006-08-25

**Authors:** David T Redden, Jasmin Divers, Laura Kelly Vaughan, Hemant K Tiwari, T. Mark Beasley, José R Fernández, Robert P Kimberly, Rui Feng, Miguel A Padilla, Nianjun Liu, Michael B Miller, David B Allison

**Affiliations:** 1 Department of Biostatistics, Section on Statistical Genetics, University of Alabama at Birmingham, Birmingham, Alabama, United States of America; 2 Clinical Nutrition Research Center, University of Alabama at Birmingham, Birmingham, Alabama, United States of America; 3 Department of Nutrition Sciences, University of Alabama at Birmingham, Birmingham, Alabama, United States of America; 4 Division of Clinical Immunology and Rheumatology, Department of Medicine, University of Alabama at Birmingham, Birmingham, Alabama, United States of America; 5 Division of Epidemiology, University of Minnesota, Minneapolis, Minnesota, United States of America; The Jackson Laboratory, United States of America

## Abstract

Individual genetic admixture estimates, determined both across the genome and at specific genomic regions, have been proposed for use in identifying specific genomic regions harboring loci influencing phenotypes in regional admixture mapping (RAM). Estimates of individual ancestry can be used in structured association tests (SAT) to reduce confounding induced by various forms of population substructure. Although presented as two distinct approaches, we provide a conceptual framework in which both RAM and SAT are special cases of a more general linear model. We clarify which variables are sufficient to condition upon in order to prevent spurious associations and also provide a simple closed form “semiparametric” method of evaluating the reliability of individual admixture estimates. An estimate of the reliability of individual admixture estimates is required to make an inherent errors-in-variables problem tractable. Casting RAM and SAT methods as a general linear model offers enormous flexibility enabling application to a rich set of phenotypes, populations, covariates, and situations, including interaction terms and multilocus models. This approach should allow far wider use of RAM and SAT, often using standard software, in addressing admixture as either a confounder of association studies or a tool for finding loci influencing complex phenotypes in species as diverse as plants, humans, and nonhuman animals.

## Synopsis

In recent years, scientific efforts to find genes influencing disease and health-related traits have sought to capitalize on the unique genetic characteristics of admixed populations. Admixture can refer to the event of two or more genetically diverse populations intermating and producing an admixed population. Admixture creates the potential for efficient identification of trait-influencing genes. However, genetic association studies using admixed populations are also prone to incorrectly concluding that a gene is linked and associated with a trait even when it is not. Several researchers have produced promising statistical methodologies for genetic association studies within admixed populations. In this paper, the authors show how these statistical methods can be unified in a broadly applicable regression framework and discuss which variables should be included in the regression models for valid testing. Because the variables required in this regression framework can only be measured with error, the authors show the consequences of these measurement errors and present measurement error correction methods applicable to this problem. By recasting the statistical methods for genetic association studies within admixed populations as regression models, a broader range of modeling and hypothesis testing becomes available.

## Introduction

When two or more populations have been separated by geographic or cultural boundaries for many generations, differential selection pressures, drift, and spontaneous mutations may lead to different allele frequencies in each population. If individuals from these founding populations subsequently mate, disequilibrium among linked markers in their offspring may span a greater genetic distance than typically found in panmictic populations. This extended disequilibrium can greatly facilitate the ability to detect regions of the genome harboring phenotype-influencing loci by reducing both the number of marker loci required and the cost when compared to disequilibrium mapping in panmictic populations [1,2]. However, this admixture process can, under some circumstances, produce disequilibrium between pairs of unlinked loci, creating confounding (i.e., spurious associations) in genetic association studies [3–5].

Recently, with the availability of genome-wide markers, the wider use and application of Bayesian statistical methods, the use of Markov chain Monte Carlo and hidden Markov methods, and the insight of several investigative groups [6–12], the opportunity for sophisticated admixture mapping has become a reality. These advances also provide the ability to control for possible confounding due to disequilibrium between pairs of unlinked loci created by the admixture process. Several strategies have been proposed for estimating admixture for individuals over the whole genome, as well as in specific regions of the genome [8,10,13]. Methods referred to as structured association tests (SATs) have been proposed that use individual admixture estimates to perform tests of association within admixed populations [7,11,14,15]. Regional admixture mapping (RAM) methods use genome-wide admixture estimates and region-specific admixture estimates to identify specific regions of the genome harboring loci that influence phenotypes [1,13,16]. These methods are especially interesting due to their potential for identifying genetic variants contributing to diseases or phenotypes that have markedly different distributions among breeding groups (or in humans, ethnic groups) [17]. Other methods, such as genomic control, proposed by Devlin and Roeder, attempt to correct for population stratification due to admixture in association testing without inferring or utilizing the details of the population structure [14,18,19]. These methods do not involve the estimation of individual admixture values and will not be discussed in detail here; however, they have been discussed and compared with existing SAT methods elsewhere [14,20,21].

The overall aim of this paper is to provide a general model that conceptually unites RAM and SAT methodologies into an extensible form. To accomplish this, we provide an overview of the problem and existing methods, followed by methodologic clarification. We then present our model and illustrate its properties via simulation. These simulations are not meant to provide comprehensive description of the operating characteristics of the methods across many situations, but rather offer illustrations of key methodological points.

## Results/Discussion

Before presenting a unifying approach, we review the justification and underlying principles of both methods.

### What Is SAT?

Hoggart et al. (p. 1492 in [7]) articulated the rationale behind SAT: “In general, population stratification exists when the total population has been formed by admixture between subpopulations and when admixture proportions (defined as the proportions of the genome that have ancestry from each subpopulation) vary between individuals. …If the risk of disease varies with admixture proportions, this will confound associations of disease with genotype at any locus where allele frequencies vary between subpopulations. …If the confounder—admixture proportions—can be measured accurately, control for it can be achieved in a straightforward manner by modeling its effects in the analysis.”

We will show that how one attempts to control for parental ancestry is critical to determining whether one eliminates potential confounding due to variations in parental ancestry. To our knowledge, there are four published approaches to SAT [7,11,12,22]. All are built on this general principle, but take somewhat different approaches. We will not explore the specifics of those approaches here but note that none are couched in a general framework that includes both RAM and SAT. Furthermore, none allow flexible generalization to as broad a range of situations as we would wish.

**Figure 1:**
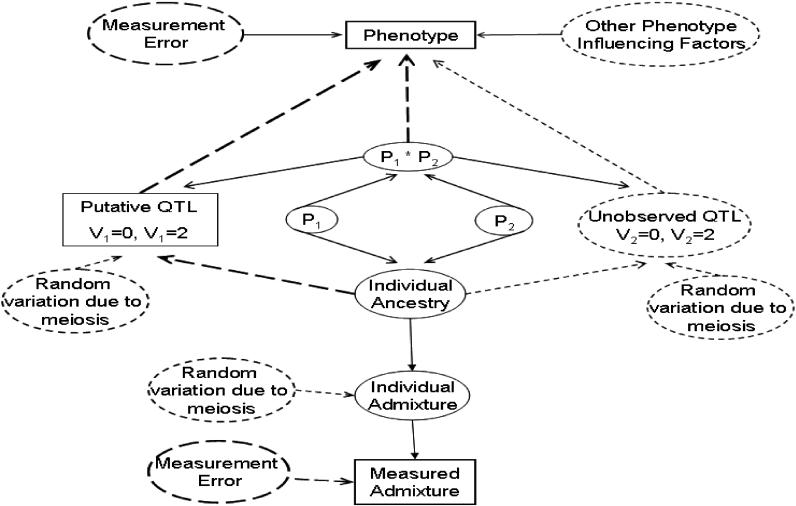
Path Diagram Illustrating the Relationship between Admixture, Ancestry, and Phenotype This figure was created based on the rules of path diagrams outlined in [65] with minor modifications. We wish to explore the association of the putative QTL with a given phenotype. However, as illustrated, this zero-order (i.e., unadjusted) association may be affected by relationships with other factors. The rectangles and ellipses in the path diagram represent observable and latent (unobservable) variables, respectively. The dashed ellipses indicate variables potentially capable of influencing the phenotype. Sources of error from random variation introduced by the meiosis process or measurement error are indicated for observable and unobservable variables. The variable *v*_i_, *i* = 1,2 denotes the number of alleles inherited from a specific parental population at the i^th^ QTL (the putative QTL, QTL 1, is observed, whereas QTL 2 is unobserved). Note that for a specific QTL, only two possible values of *V*_i_, *i* = 1,2 are considered in the model; the third possible value will serve as a reference. *P*_i_, *i* = 1,2 represents the ancestry of each parent for a sampled individual. The objective is to test for association between the putative QTL and the observed phenotype. Observed association may simply result from unaccounted correlation among the putative QTL, the phenotype, and individual ancestry. The association can be further confounded by the presence of unobserved factors, such as QTL 2. Controlling for parental and individual ancestry would break this confounding pathway. Because ancestry is not directly observable, individual admixture estimates are used as surrogates. These estimates, obtainable through existing software, can be seen as error-contaminated measurements of the true individual ancestry values. Hence, the measurement error problem must be addressed when including these estimates in the model. Hoggart et al. [7] offer a figure similar to the one presented here.

The overall issue of confounding due to admixture disequilibrium, generalized to any population, is portrayed in the path diagram of Figure 1. In the path diagram, rectangles represent directly observed variables, ellipses represent unobserved or latent variables, dashed ellipses represent variables that can potentially exert influences, and arrows represent direct or casual relationships. The path diagram introduces two key latent constructs, individual ancestry and individual admixture, which underlie the issue of confounding due to variation in individual ancestry. Specifically, an individual ancestry proportion, with respect to a specific parental population, is defined as the proportion of that individual's ancestors who were members of that parental population in the generation prior to the first admixture event. This is in contrast to an individual's admixture, which is the proportion of the individual's genome that is inherited from a specific parental population.

The figure indicates that association testing is not a simple issue. The relationship between the putative quantitative trait locus (QTL) and phenotype is the one of interest, but it can be confounded by other variables. First, note that QTLs and individual admixture can be directly influenced by random variation due to meiosis. In addition, both the phenotype and measured admixture are potentially subject to measurement error. Furthermore, measured admixture is directly affected by individual admixture, which in turn is affected by individual ancestry. Naturally, the ancestry of the parents, represented by P_1_ and P_2_, affects individual ancestry. Individual ancestry can directly affect the putative QTL, which in turn can affect the phenotype, so individual ancestry has an indirect affect on the phenotype via the putative QTL. The right–hand side of the path diagram is a mirror image of the left–hand side, with unobserved QTL replacing the putative QTL and represents the potential path of spurious associations. The diagram also indicates that the product of parental ancestries also affects both QTLs. Justification for these paths is provided below.

**Figure 2:**
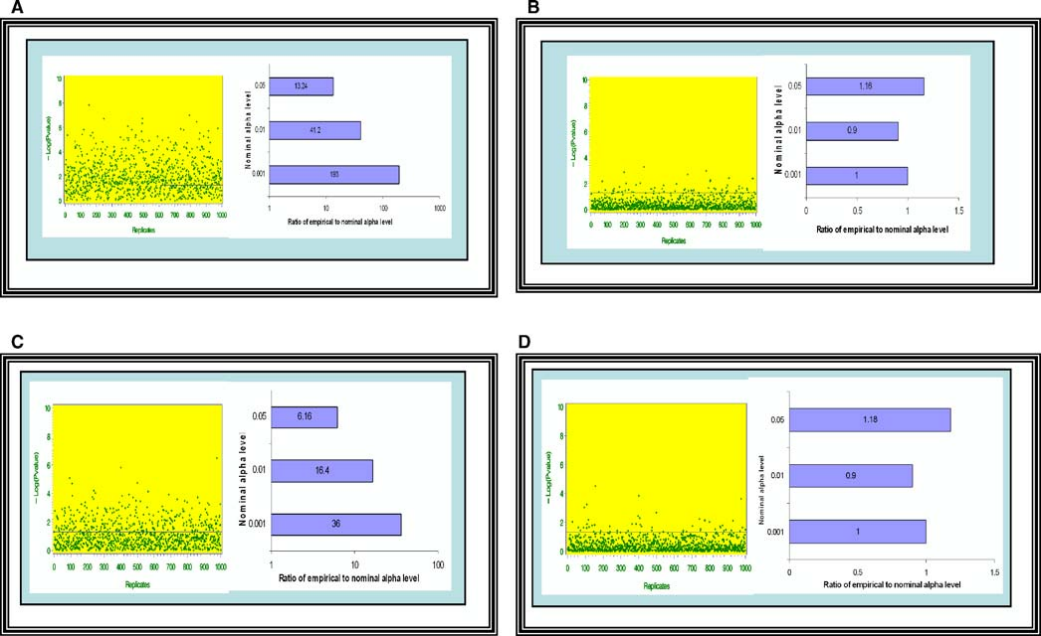
Conditioning on Individual Ancestry and the Product of Parental Ancestries Is Necessary and Sufficient to Control for Confounding A dataset was simulated from idealized circumstances for the purposes of illustration. The dataset contained 1,000 individuals that were admixed from parental populations *V* and *V*. For each individual, both parents had the same amount of *V* ancestry. The *V* ancestry proportion of each individual was drawn from a beta distribution (Beta [0.3771, 0.8341]). These parameter values were based on estimates of African ancestry proportions from a sample of 479 individuals recruited from different previously described studies in New York City, New York, and Birmingham, Alabama [66–68]. We simulated a trait-influencing diallelic QTL (G1) that had alleles G and g with frequencies 0.2 and 0.8, respectively, in population *V* and frequencies 0.8 and 0.2, respectively, in population *V*. We simulated a phenotype, Y, that was a function of G1 and a random normal deviate. Finally, we simulated a marker (G2) that had alleles with frequencies 0.2 and 0.8 in population *V* and complementary frequencies in population *V*. Alleles at G2 did not influence Y and G2 was unlinked to G1. However, G1, G2, and Y are all correlated with ancestry. However, the association between G2 and Y is spurious. We then test for association between Y and G2 by regressing Y on two dummy codes for the genotypes at G2 [69] and conducting a two degrees of freedom (df) test under the following scenarios: (1) without any type of control (i.e., no covariates); (2) controlling for linear term of true individual ancestry when the alleles at G1 act in an additive fashion; (3) controlling for linear term of true individual ancestry when the alleles at G1 act in an overdominant fashion; and (4) controlling for linear and quadratic term of true individual ancestry when the alleles at G1 act in an overdominant fashion. Because we imposed the simplifying condition that for each individual, both parents had the same amount of *V* ancestry, the square of individual ancestry is equivalent to the product of parental ancestries. Since alleles at G2 do not cause variation in Y nor is G2 linked to a gene that causes variation in Y, every significant association found under any of the above scenarios constitutes a false positive. The graphs in this panel were created by simulating 1,000 independent replicate datasets. The dots on each graph located on the left portion of each panel represent the observed *p* values (expressed on a −log_10_ scale) for the test for the effect of G2 for each dataset. The bar plot of the right section of each panel represents the observed ratio of the empirical to the nominal type I error for each simulation. (A) Not controlling for ancestry leads to inflated type I error. The degree of type 1 error rate inflation increases with smaller α levels. (B) Controlling for only the linear term of individual ancestry is sufficient only when the confounding QTL affects the phenotype only in an additive fashion. In this case, there was no excess of type 1 errors. (C) When the QTL affects the phenotype in a nonadditive fashion (in this case, through overdominance), controlling for the linear term of ancestry is insufficient to remove the confounding effect. The type I error rates remain quite inflated even after including true individual ancestry in the model. (D) When the QTL affects the phenotype in an overdominant fashion, controlling for true individual ancestry and the product of parental ancestries effectively eliminates the confounding. In this case, the ratios of empirical to nominal α levels are within sampling error of 1.0.

The consequences of failing to control for variation is ancestry is illustrated in Figure 2A. The simple simulation reveals type I errors occur 13.24, 41.2, and 193 times as often as expected at the .05, .01, and .001 α levels, respectively, and this inflation is attributable to confounding due to variation in ancestry. SATs are designed to be resistant to such confounding.

### What Is RAM?

We define region-specific admixture as a characteristic of segments of the genomes of individuals. For any given region of the genome, one's region-specific admixture from population *V* is the proportion of alleles in that region that are copies of alleles from members of population *V.* The rationale for RAM rests on two premises. First, the process of admixture creates linkage disequilibrium among linked loci that tends to extend over longer genetic distances than does disequilibrium under long-term panmixia. Second, even after appropriately adjusting for the degree of individual ancestry, the degree of individual region-specific admixture will covary with phenotypes that are influenced by loci that are (1) in the region under study; and (2) in disequilibrium with loci that have different allele frequencies in the parental populations. Both premises are well established [23,24]. Prior to the late 1990s, several authors had formally discussed the possibility of RAM-type approaches [23], but did not offer methods that would control for potential spurious associations [4]. McKeigue first introduced modern approaches to RAM that attempted to control for spurious associations induced by the admixture process [6,25,26].

Several approaches to RAM [6,13,16,25–29] have been published. Some [28] use a two-stage approach in which estimates of individual admixture and region-specific admixture are first obtained in a specialized procedure and then used in an ordinary logistic regression approach with case-control data. This two-stage approach lends itself to generalization and is a simplified form of the unified general linear model approach we present.

### Methodologic Clarifications

There are a number of methodologic points that have been alluded to but have not been completely elucidated in the literature pertaining to how one should condition upon (control for) ancestry within RAM and SAT. Within the next few sections, we seek to clarify these points.

It is unclear from past writing whether it is sufficient to control for individual admixture, individual ancestry, or both to eliminate confounding due to the admixture process. We first clarify that, although sometimes used interchangeably, an individual's admixture and an individual's ancestry are not equivalent variables. To illustrate, consider a set of full siblings that does not include any monozygotic twins. Because they are full siblings, all individuals in the set have equal individual ancestry from specific populations or regions. In fact, all individuals in the set have ancestry equal to the mean or midpoint of their parent's ancestries, represented as P_1_ and P_2_. However, due to recombination, all individuals will have slightly different admixture values.

Here we show by counterexamples that it is not sufficient to control for individual admixture and it is also not sufficient to control for individual ancestry. We then show that it is sufficient to control for both individual ancestry and the product of parental ancestry. Throughout the paper and our examples, *i* represents the i^th^ individual, *j* the j^th^ locus, *k* the number of alleles at the j^th^ locus, and *V* the number of founding populations. For simplicity we assume 2 founding populations in this paper.

#### Controlling for individual admixture is not sufficient.

Given variations in parental ancestry, controlling for individual admixture is not sufficient. Imagine an organism with *W* independent genetic segments of equal genetic length. For each individual, let the two parents have equal ancestry. Suppose that the admixture of each segment is known without (measurement) error. Without loss of generality, assume that the segment-specific admixture values (denoted *X_j_* for the j^th^ segment) and the ancestry values are all scaled to have variance 1.0. Given the assumptions above, all segment-specific admixture values will have equal covariance with ancestry. Denote this covariance as β*.* Let

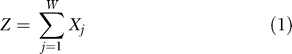
denote the overall individual admixture value (for ease of exposition, we have not divided by *W,* but this is only a linear transformation and will have no impact on the result). Then, the correlation coefficient between *X_j_*_1_ and *X_j_*_2_ is 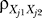 = β^2^. The correlation coefficient between *X_j_*_1_ and *Z* is

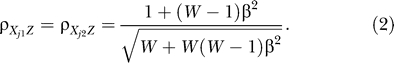



The correlation coefficient can be written in terms of simple correlation coefficients

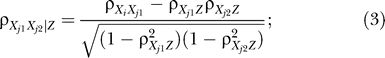
after substituting and reducing,

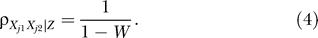
for *W* > 1. Thus, it is clear in this situation that the partial correlation coefficient can never be zero and only asymptotically approaches zero as *W* approaches infinity (i.e., as the amount of independent information that goes into the emergent variable of admixture increases infinitely). If 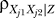 is not guaranteed to be zero, then, conditional on individual admixture, what is inherited at one segment can be correlated with what is inherited at another chromosome. Therefore, controlling for individual admixture is not sufficient to eliminate correlations among unlinked loci and is not sufficient to control for spurious associations. The formula further implies that the distinction between individual ancestry and individual admixture will, all other things being equal, be greatest in organisms such as *Arabidopsis* (diploid chromosome number = 10, 8.0 × 10^7^ base pairs in total length) with short genomes and less in organisms such as crayfish (diploid chromosome number = 200, 8.22 × 10^9^ base pairs in total length) with long genomes (see http://www.genomesize.com).

#### Controlling for individual ancestry is not sufficient.

Let *X*_1_ and *X*_2_ denote Bernoulli-distributed random variables indicating whether or not one has inherited two alleles from population *V* at locus 1 and locus 2, respectively, and let *v_ij_* denote the number of alleles inherited from population *V* at the j^th^ locus for the i^th^ individual. Assume that the two loci are unlinked and that we begin with two inbred populations, *V* and V̵ (not *V*), denoting nonadmixed individuals from population *V* as *VV* and nonadmixed members of the population V̵ as V̵ V̵ . Subsequently, *N*_1_ and *N*_2_ individuals from populations *V* and V̵ , and, subsequently their offspring, begin intermating for two generations in an unspecified pattern. Then, in the second admixed generation, we have a population that can be described as in Table 1.

**Table 1:**
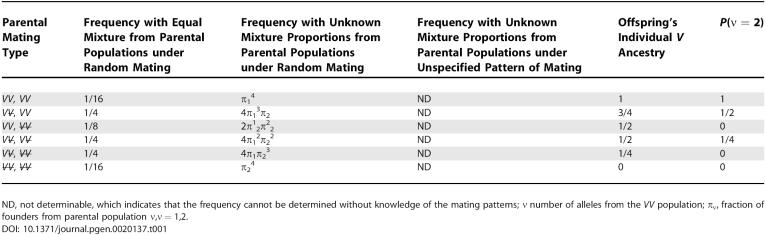
Expected Population Resulting from Two Generations of Random Mating between Two Inbred Populations

As can be seen in Table 1, *P*(*v_ij_* = 2) is not determined solely by individual ancestry but also depends on mating patterns and mixing proportions, via their influence on the distribution of parental mating types. This means that, even conditional upon individual ancestry, there can still be confounding because *X*_1_ will be correlated with *X*_2_ *.* Controlling for individual ancestry may remove most of the confounding, but not all. This is even more evident when one imagines a dataset including only the two rows with *V* ancestry of 1/2. Within these two rows, although individual ancestry would be controlled perfectly (there would be no variation), the opportunity for confounding is present. Only members of the *V* V̵ × *V* V̵ matings can have either *X*_1_ = 1 or *X*_2_ = 1.

Some models (e.g., [7,12]) control for the linear effect of individual ancestry or individual admixture in regression-type models in an attempt to insure that RAM and SAT tests are not confounded by variation in ancestry. This will only be valid if one tests only for linear allelic (additive) effects at loci without testing for dominance (genotypic) effects or epistasis. This is because when testing for the allelic effects, the expected number of alleles from population *V* at any one locus among individuals with ancestry *A* from population *V* is

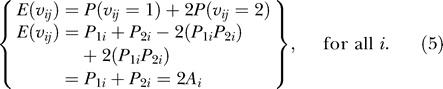



However, the locus-specific effects on complex and quantitative traits cannot a priori be assumed to be additive and can even be overdominant [30–34]. For this reason, many investigators wisely choose to test for genotypic effects in two degrees of freedom models (e.g., [12]) rather than restricting themselves to allelic (additive) effects (compare with [35]). In such situations, controlling only for the linear term of individual ancestry will be insufficient if one uses tests that allow for nonadditive genotypic effects.

#### Controlling for individual ancestry and the product of parental ancestries is sufficient.

The premise of conditioning on parental ancestry was first introduced by McKeigue [26]. Here we expand on the idea and show that it is necessary to condition on both individual ancestry and the product of parental ancestries. It is important to note in the following that, although we are controlling for parental ancestries, this does not imply it is necessary to include parents in RAM and SAT studies (see Text S1 for discussion of estimating parental ancestry solely from offspring data).

Let *P*_1*i*_ and *P*_2*i*_ denote the individual ancestries from population *V* for the two parents, respectively. Note that for any locus, the expected number of *V* alleles depends only on the individual's ancestry; hence, we drop the locus-specific subscript *j* in subsequent equations. Then, at every locus:

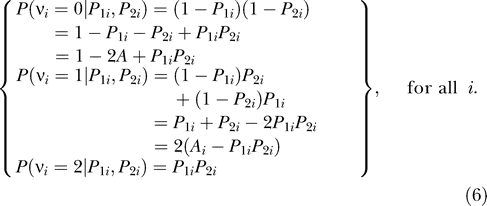



Furthermore, conditional on *P*_1*i*_ and *P*_2*i*_ *,* the number of alleles inherited from one population at a given locus is independent of the number of alleles inherited at another locus for all loci that are unlinked as defined by Mendel's law of independent assortment. Therefore, controlling for *P*(*v_i_* = 0 | *P*_1*i*_, *P*_2*i*_), *P*(*v_i_* = 1 | *P*_1*i*_, *P*_2*i*_), and *P*(*v_i_* = 2 | *P*_1*i*_, *P*_2*i*_) is sufficient to eliminate confounding by unlinked loci. Given that *P*(*v_i_* = 0 | *P*_1*i*_, *P*_2*i*_) + *P*(*v_i_* = 1 | *P*_1*i*_, *P*_2*i*_) + *P*(*v_i_* = 2 | *P*_1*i*_, *P*_2*i*_) = 1, it is only necessary to control for any two in a model. We choose to control for *P*(*v_i_* = 0 | *P*_1*i*_, *P*_2*i*_) and *P*(*v_i_* = 2 | *P*_1*i*_, *P*_2*i*_). If we let *Y* denote a phenotype and *f*(*Y_i_*) denote some function of *Y,* then a testing model that would eliminate confounding induced by variations in parental ancestry would take the form:

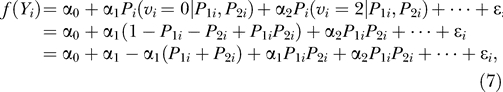
in which the missing terms denoted by the ellipsis are those that one is primarily interested in testing. Letting β_0_ ≡ *α*_0_ + *α*_1_, β_1_ ≡ −2*α*_1_, and β_2_ ≡ *α*_1_ + *α*_2_ and substituting terms yields:

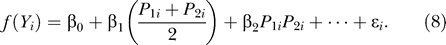
Noting that, by definition, (*P*_1*i*_ + *P*_2*i*_)/2 is individual ancestry (*A_i_*), yields:

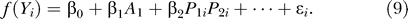
As can be seen, the probability distribution of the descent status (and therefore the genotypes if allele frequencies differed in the parental populations) depends on both first- and second-order functions of ancestry but not on any higher-order terms. Thus, to eliminate confounding due to variations in parental ancestry, it is sufficient to control for individual ancestry and the product of parental ancestries. Figure 2B–2D illustrates these points. Specifically, Figure 2B indicates that if the confounding locus acts in a additive fashion, controlling for ancestry without the product of parental ancestries does provide adequate type I control. However, Figure 2C reveals type I errors occur 6.16, 16.4, and 36 times as often as expected at the .05, .01, and .001 α levels, respectively, when the confounding locus acts in an overdominant fashion and the linear term of ancestry alone is used to control for variation in ancestry. Finally, Figure 2D indicates adequate control is achieved when the confounding locus acts in an overdominant fashion and both the linear term of ancestry and the product of parental ancestries are used to control for variation in ancestry.

#### The insufficiency of “conditional conditioning.”

**Figure 3:**
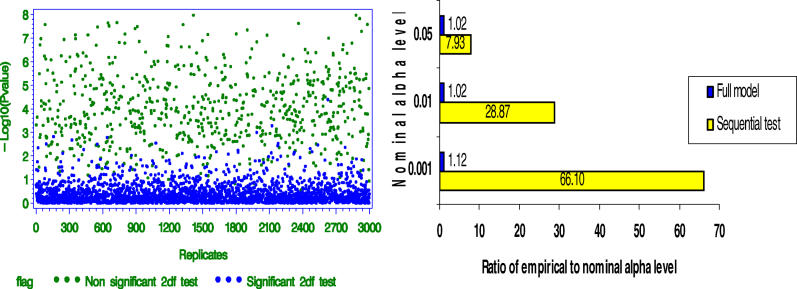
Effect of “Conditional Conditioning” on Type 1 Error Rates We simulated datasets containing a phenotype Y that is associated with a marker G_1_ and true ancestry. We also simulated another marker G_2_ that is not associated with Y, but like Y, is correlated with true ancestry. Therefore, any significant association between Y and G_2_ is considered a false positive. We consider the full model *Y* = β_0_ *+* β_1_ *A_i_ +*β_2_ *P*_1*i*_ *P*_2*i*_ *+* β_3_ *G*_2_ + ɛ*_i_*. We begin by testing the null hypothesis H_0_: β_1_ = 0 and β_2_ = 0. If this test is significant, the p-value represented by the blue dots is obtained from the full model, otherwise we obtained the *p* value (green dots in the graph) from the restricted model *Y* = β_0_ *+* β_3_ *G*_2_ *+* ɛ*_i_*. As can be seen, *p* values tend to be quite small when we do not include the nonsignificant terms in the final model. The bar graphs on the right hand side show the type I error inflation (yellow bars) when one tests for association between Y and g_2_ in a sequential fashion; that is by first testing H_0_: β_1_ = 0 and β_2_ = 0, and relying on the outcome of this test to decide whether to control for ancestry. The correct α levels are obtained by always including ancestry terms in the model regardless of their levels of significance.

One may choose to condition on parental ancestry only if parental ancestry is found to be statistically significant when included in the model or if significant structure is detected in the sample as was described by Pritchard et al. [22] as the first step in their three-step SAT procedure and by Hoggart et al. (p. 1502 in [7]). We refer to this approach as conditional conditioning. If one's goal is to ensure that under H_0_, the type 1 error rate remains ≤ α, which generally defines a valid test in the frequentist context, then conditional conditioning is not a valid testing strategy. That is, even though covariates may not meet criteria for statistical significance in a finite sample, this does not mean they are not confounders, and failing to include them in the model can lead to inflated type 1 error rates [36]. Therefore, if one is interested in valid RAM and SAT tests of linkage in the presence of association, it is necessary to control for parental ancestry terms as in Equations 10 and 11 regardless of their degree of statistical significance in the model. By analogy, the practice of only controlling for parental ancestry only if a significance test of Hardy-Weinberg equilibrium is rejected has the same problem [37]. So too would the practice of attempting to control for parental ancestry only if other tests yielded significant evidence that the sample came from a structured population. This is illustrated in Figure 3 **,** which reveals type I errors occur 7.93, 28.87, and 66.1 times as often as expected at the .05, .01, and .001 α levels, respectively, when conditional conditioning is used.

### A General Linear Model

Here we introduce general models for RAM and SAT that are highly extensible. We define the following notation: *Y,* a phenotype that can be continuous, ordinal, or dichotomous; *A_i_,* ancestry for the i^th^ individual, the proportion of the i^th^ individual's ancestors that came from parental population *V*; *A_ijk_,* a dummy-coded (0,1) indicator variable indicating whether the i^th^ individual has inherited *k* and only *k* alleles at the j^th^ locus from an ancestor that was from parental population *V*; and *G_ijk_,* a dummy-coded (0,1) indicator variable indicating whether the i^th^ individual has *k* and only *k* alleles at the j^th^ locus of a specified type. We use *f*(*Y_i_*) to denote the link function, a monotone function linking the dependent variables to the estimated model [38], a device also employed by Hoggart et al. [7]. We offer the following simple models for generalized RAM and SAT. We assume for now that all variables are known without error. However we return to the important issue of measurement error issues later.

RAM model:


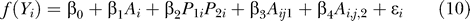


SAT model:


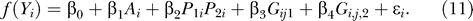


These general linear models are very flexible. First, dichotomous (e.g., case vs. control), ordinal, time-to-event, or continuous phenotypes can be accommodated by letting the regression model be logistic, Poisson, Cox, or ordinary least squares, respectively. This flexibility is important. Investigators frequently want to not only assess genetic association for dichotomous and static phenotypes such as lupus (yes vs. no) in a case-control study, but also wish to assess genetic association with longitudinal outcomes (e.g., clinical course in medical research or growth rate in agricultural research), adjusting for covariates including demographic and ancestry. Such longitudinal phenotypes can also be accommodated by this general model via the use of mixed models and related techniques for longitudinal data [39,40]. Therefore, the models can be fit in standard software (e.g., SAS), which has the advantage of being widely accessible, well documented, and well tested. This radically increases the likelihood of wide and proper use. Moreover, by being framed in a regression approach, all of the machinery of regression, including diagnostics [41], well-recognized effect size metrics, robust variations [42], the ability to include covariates, and the ability to test interactions are at one's disposal. This immediately makes the models extensible to multilocus and epistatic models. Finally, the RAM approach can be expanded to test a region of a chromosome by, instead of including marker-specific ancestry, including an estimate of the admixture of the region.

#### A conceptual bridge to identity in state and identity by descent.

Another advantage of the models in Equations 10 and 11 is that they make clear the relationships between RAM and SAT and identity by descent and identity in state in family-based tests of linkage and linkage in the presence of association. RAM is analogous to linkage testing, whereas SAT is analogous to association testing. The *A_ijk_* values correspond to “descent states,” whereas the *G_ijk_* values correspond to specific allele states. Indeed, Zhu et al. [16], citing [26], refer to such *A_ijk_* quantities as “X by descent” to denote an allele having ancestry from X. This conceptual bridge is more than an intellectual nicety. It immediately makes clear how we can borrow the concept of testing for linkage conditional upon association that is now popular in linkage analysis [43–45], as we shall discuss below.

### Model Extensions

As already discussed, the models in Equations 10 and 11 are easily extended to allow for any phenotypic distribution. Because no constraints are placed on the distribution of the phenotypes, with two exceptions, the models can accommodate selective sampling (e.g., sampling phenotypically extreme subjects or sampling subjects on the basis of ancestry) without modification. In addition, covariates, multiple loci, gene by environment (or gene by sex, gene by age, etc.), and gene by gene (epistasis) effects are easily modeled by simply adding appropriate terms to the right side of the equation. The general linear model presented here can be extended to deal with several situations, which are briefly introduced below. If there are a total of *M* phenotypes to include, one can replace the variable *Y* on the left side of Equations 10 or 11 with a weighted linear composite of *Y* values representing the multiple phenotypes as follows:

Multivariate RAM model:


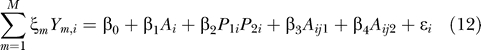


Multivariate SAT model:


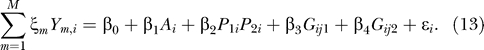


The ξ*_m_*s are constants to be estimated within the regression framework and are constrained such that 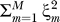 = 1. This constraint is necessary to make the model identifiable.

To our knowledge, no current RAM or SAT test allows related individuals to be included as subjects. (We distinguish the inclusion of related individuals as subjects from the requirement that parents or other relatives be included in some testing procedures as a means of controlling for ancestry [e.g., [46,47].) Equations 10 and 11 can accommodate related individuals by utilizing software that models the covariance structure among the residuals. Finally, proper estimation of parental ancestry values will require special accommodations for related individuals (e.g., full siblings should obviously be constrained to have the same parental ancestry values, etc.).

The general linear model offered can be extended to allow one to test for linkage conditional upon association with a polymorphism in a region and, thereby, test whether that polymorphism appears to account for an observed linkage signal that was detected with RAM. The right side of Equation 10 can be expanded to include the *G_ijk_* values. In this situation, one desires a test of whether the amount of variance explained by the *A_ijk_* variables conditional on all other variables in the model is significantly less when the *G_ijk_* values are included in the model compared to when the *G_ijk_* values are excluded from the model. In many cases, these tests entail the use of bootstrapping.

### Nonparametric Measurement Error Assessment and Accommodation

Until now, we have assumed that all variables are known without error. In reality, this will not be the case and is an important point to recognize. Any of the variables involved can be measured with error and we now address the consequences of error in each and propose responses to ensure validity of the tests in terms of type 1 error rate control. Throughout, we assume that the measurement errors are independent of each other and of all of the variables under study. We also do not dwell on how one should calculate estimates of individual and parental admixture or estimates of the reliability thereof when used as estimates of individual and parental ancestry. For now, we simply assume that it is possible to do so and briefly address ways in which this might best be accomplished in the Text S1.

#### Error in the genotypes.

It is well known that genotyping errors occur and, when they occur, result in reduced power [48]. However, if the measurement error is in the determination of *G_ijk_,* this will only lower power, not inflate the type 1 error rate. Therefore, no response is needed to ensure validity of the test.

#### Error in the phenotypes.

Phenotypes are also often measured with error but, again, this will only serve to lower power of the tests we offer and not inflate type 1 error rates [49]. Therefore, no response is needed to ensure validity of the tests.

#### Error in the estimates of region-specific individual admixture.

Unless a perfectly informative marker (i.e., a marker with allele frequencies of zero and one in one parental population and complementary frequencies in the other, respectively) is available at exactly the locus under study, the degree of regional admixture for any individual will only be known probabilistically. Let us denote the (Bayesian posterior) probabilities of individual region-specific admixture as:

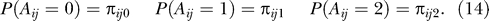
Then one can replace *A_ij_*_1_ and *A_ij_*_2_ with *π_ij_*_1_ and *π_ij_*_2_, respectively, in the various regression models in an analogous manner to what would be done in some multipoint mapping approaches in experimental crosses (see p. 433 in [50]). Measurement errors here will, again, lower power, but not affect the type 1 error rate.

#### Error in the estimates of parental ancestry.

**Figure 4:**
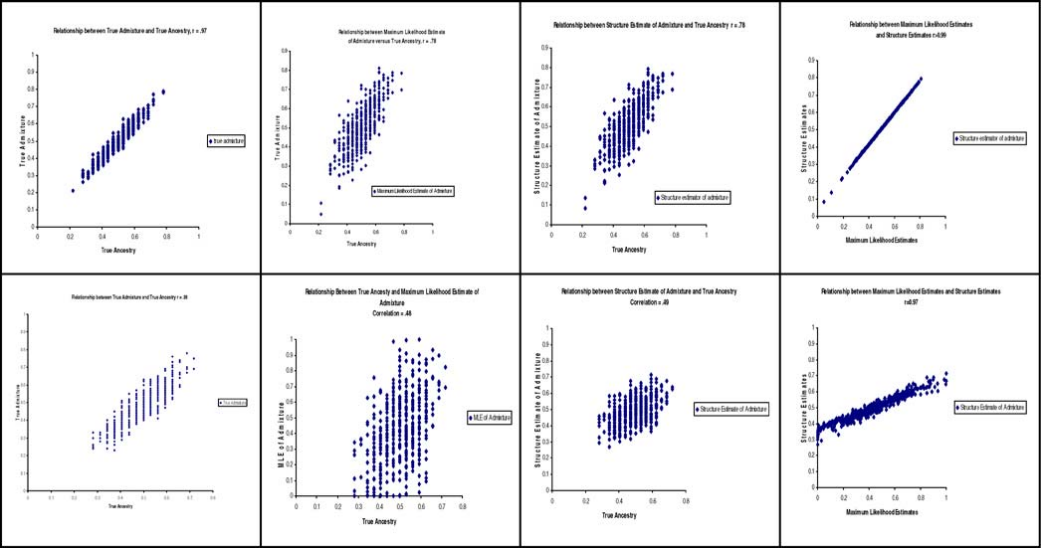
Reliability of Individual Admixture Estimates Used as Estimates of Individual Ancestry We simulated a randomly mating population or organisms based upon the “island model” or intermixture admixture process [16]. Because the data are simulated, true individual ancestry and true individual admixture are known for each individual. True individual ancestry is displayed on each abscissa. The top four panels each contain data from a simulation of 500 admixed individuals five generations after the admixture event. Two hundred ancestry informative markers are genotyped with an average allele frequency difference between the original parental populations of 0.3. Founders (250 from each parental population) were simulated for use in the procedures that estimated individual admixture. The bottom four panels also each contain data from a simulation of 500 admixed individuals five generations after the admixture event. However, here only 50 ancestry informative markers are genotyped with an average allele frequency difference between the original parental populations of only 0.2 and only 40 founders (20 from each parental population) were simulated for use in the procedures that estimated individual admixture. Maximum likelihood estimates were calculated using Tang et al.'s [10] method. Structure estimates were produced using software described here [8,64]. Several points are noteworthy. First, our results in the top and bottom rightmost panels recapitulate results obtained by Tang et al. [10] and Zhu et al. [16]. However, our results also show that even though two methods of estimating individual admixture may produce correlations very close to 1.0, the correlation of these estimates with true ancestry may be far lower (only ~.80 in our upper row and only ~.50 in our lower row). Finally, the two leftmost figures highlight the fact that there are important differences between true admixture and true ancestry.

Error in the estimates of parental ancestry poses the greatest challenge. As several authors [7,13] noted, unchecked errors in the putatively confounding variables on which one must condition will lead to incomplete control and potentially to residual confounding [51]. Therefore, some method is required to deal with measurement error in the estimates of individual ancestry. Moreover, such measurement errors, or unreliability, can be substantial, as it is illustrated in Figure 4.

Montana and Pritchard [27] noted that Hoggart et al. [7] had criticized their use of a two-stage approach in which one first calculates ancestry estimates and then in a separate analysis uses those estimates as covariates. A basis of the criticism was that this approach does not account for uncertainty (measurement error) in the ancestry estimates. Montana and Pritchard (p. 786 in [27]) acknowledge that this concern is “theoretically plausible, [but that] extensive simulations of the admixture mapping tests presented here, as well as simulations of the STRAT test … show that, in practice, the statistical tests are indeed correctly calibrated under the null hypothesis… [and that] there are some practical advantages to the two-stage process. First, the two-stage process makes the output much more transparent and interpretable for the end user. Second, it makes it much easier for users to take the ancestry estimates and develop other tests of association that are appropriate for their own data.” We agree with Hoggart et al. [7] that the measurement errors are a concern and our simulations herein demonstrate that under some circumstances measurement errors can produce substantial type 1 error rate inflation. On the other hand, we also agree with Montana and Pritchard [27] that the advantages of the two-stage approach in terms of flexibility and conceptual clarity are profound. Fortunately, measurement error correction methods can allow “the best of both worlds” by retaining the flexibility of the two-stage approach while properly accounting for the measurement error.

While many methods are available (e.g., [52,53]), the most common approach to dealing with errors in variables on the right side of regression equations is regression calibration. In some circumstances (e.g., linear regression), it is effectively the correction for attenuation. This method is a type of resubstitution; instead of the true but unobservable predictor, one substitutes an estimate of it, conditional on the observed covariates (but not the response). Then the idea is to run a standard analysis, and “fix up” the standard errors at the end via devices such as bootstrapping. In linear regression, regression calibration is often considered the default option because it often works surprisingly well. In logistic regression with a relatively rare disease, regression calibration is an almost exact method. One of the major advantages of regression calibration is that it is easy to implement; after the resubstitution, a standard analysis can be run to obtain estimates [54].

Another alternative is the simulation extrapolation (SIMEX) approach [54–57]. SIMEX is more computationally intensive than regression calibration, but it is one of the major default options for nonlinear models that cannot be handled by correction for attenuation techniques or regression calibration—that is, it is extremely flexible and can be used with any incarnation of the general linear model. It is also extremely useful for problems in which the measurement error is not of the classic, additive homoscedastic type, as will occur, for example, in the current case in which the predictor variable (ancestry) is a proportion. As with regression calibration, a great advantage of SIMEX is that it separates the primary statistical modeling component from the error correction component, thereby freeing data analysts to implement the full range of their usual battery of procedures.

**Figure 5:**
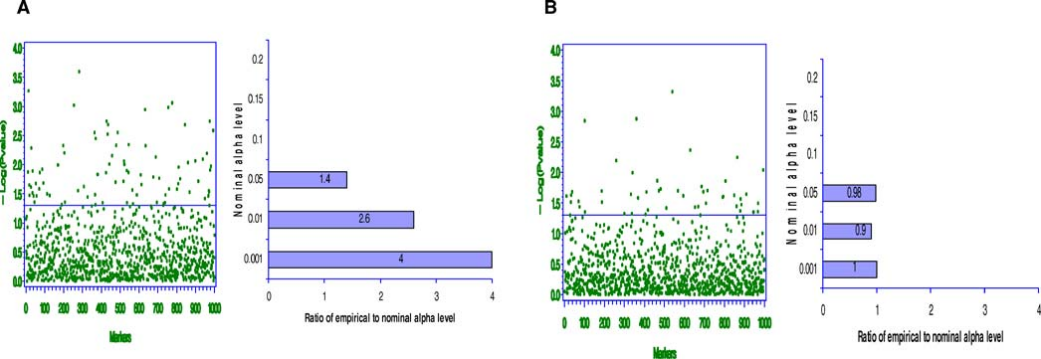
The Importance of Accommodating Measurement Error in Models The dataset used to create this graph was generated under the same conditions as used to generate the data for Figure 2. The reliability of the available individual admixture estimates used as estimates of individual ancestry is 90%. That is, {(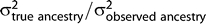 ) = [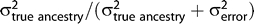 ] = 0.9}. (A) Type I error inflation caused by measurement error in the individual ancestry estimate. Ignoring possible measurement error in the ancestry estimate may also lead to a high type I error rate. (B) Observed false positive rate after correction for measurement error; in this example we used the SIMEX algorithm as described in Cook and Stefanski [70].

Several other methods exist [58], including multiple imputation [59]. Figure 5A and 5B, respectively, illustrate the residual confounding that can occur when conducting a SAT procedure without correcting for measurement error and the proper control of confounding that occurs when a measurement error correction is used. Figure 5A reveals type I errors occur 1.4, 2.6, and 4 times as often as expected at the .05, .01, and .001 α levels, respectively, when the correct SAT model is specified but imperfect measured of ancestry are used. Once measurement error corrections are applied, Figure 5B indicates that the correct type I error rates are restored.

### Future Directions

Our purpose here has not been to become bogged down in the logistics of setting up RAM and SAT studies or to provide detailed evaluations of the performance characteristics of specific designs and analytic implementations. Rather, our goal was to articulate a unified and generalizable approach to RAM and SAT. We have shown through proofs, counterexamples, and small simulations that it is necessary and sufficient to condition on both individual ancestry and the product of parental ancestries, and it is not sufficient to “conditionally condition” on parental ancestries, in order to control for confounding in admixture studies. We provide a general linear model that is extensible to a multitude of study designs, conditions, and populations of interest that are briefly presented, but left to future work for detailed descriptions. Within Text S1, we have also provided a semiparametric reliability assessment method as well as suggestions for accommodating measurement errors. It is worth noting that several open questions, or areas for future research, remain in order for studies using RAM and SAT to be optimally useful. These include expanding our RAM approach to case-only analysis, methods for selecting markers with which to estimate ancestry, development of panels of such markers for different ethnic groups (or demonstration that such a priori–defined panels are not needed [60]), and evaluation of methods for estimating individual ancestry and region-specific admixture (for further discussion on such issues, see [2,61,62]). Additional issues include how RAM and SAT can best be utilized in studies involving DNA pooling and how individual ancestry estimation procedures, and the estimation of the reliability thereof, can best utilize knowledge about the pedigree structure among individuals when related individuals are studied. How to best accommodate pedigree data in the analyses remains a question for RAM and SAT as it does for association testing in general [63]. Finally, now that a general model exists, the time is opportune for a thorough evaluation of the performance characteristics under multiple different population genetic models, genetic architectures, sampling strategies, and phenotypic distributions.

## Materials and Methods

Simulation studies were performed using the software SAS (Cary, North Carolina, United States) under the “general island” and intermixture models presented by Zhu et al. [16]. The SAT model β *f*(*Y_i_*) = β_0_ + β_1_ *A_i_* + β_2_ *P*_1i_ *P*_2i_ + β_3_ *G_ij_*_1_ + β_4_ *G_ij_*_2_ + ɛ*_i_* was used to simulate the association of admixture and ancestry with a putative QTL for different situations. Admixture estimates were provided by Structure [8,64] and Tang's maximum likelihood estimate method [10]. Further details are provided in the figure legends.

## Supporting Information

Text S1 RAM SAT (118 KB DOC)

## Abbreviations

QTL: quantitative trait loci, (or locus)
RAM: regional admixture mapping
SAT: structured association testing
SIMEX: simulation extrapolation
