# Supplementary material for: Regional Admixture Mapping and Structured Association Testing: Conceptual Unification and an Extensible General Linear Model
Source: PLoS Genet. 2006 Aug 25;2(8):e137. doi: 10.1371/journal.pgen.0020137 (PMC1557785; doi:10.1371/journal.pgen.0020137)
Supplement: Text S1 — (118 KB DOC) [file pgen.0020137.sd001.doc]

**TEXT S1 – MEASURING ADMIXTURE, ANCESTRY, AND THEIR RELIABILITY**

Our primary purpose in this paper was to address the conception and implementation of RAM and SAT under the assumption that one had a way of estimating the relevant variables and of evaluating the reliability of these estimates. Here we briefly address some of the issues in estimating those quantities and describe some approaches that might be useful. Our purpose is neither to provide detailed algorithms for such estimation nor to explore the relative performance characteristics of these approaches. Instead we aim to clarify that such estimation can be done and outline some promising, and in some cases, previously unrecognized approaches.

**ASSESSING RELIABILITY of ESTIMATES of INDIVIDUAL ANCESTRY and the PRODUCT OF PARENTAL ANCESTRIES.**

Implementing most, if not all, measurement error correction procedures requires some information about the reliability of variables measured (or estimated) with error. We define reliability as the proportion of variance in the measured values that is attributable to the true values. For generality, we refer to whatever ancestry-related variable is being estimated (whether it is individual ancestry, the product of parental ancestries, etc.) as $\theta_{i}$denoting the value of $\theta$ for the i^th^ individual and $\hat{\theta}_{i}$denoting the estimate thereof.

**Extensions of existing approaches**.

Let $\hat{\sigma}_{\hat{\theta}_{i}}^{2}$denote the estimated variance of $\hat{\theta}_{i}$. The two most common approaches used to estimate individual values of ancestry-related variables are maximum-likelihood estimation (MLE) and Bayesian Markov chain Monte Carlo (MCMC) approaches. With MLE, $\hat{\sigma}_{\hat{\theta}_{i}}^{2}$ can be estimated from the inverse of the negative second derivative of the likelihood function (i.e., Fisher’s Information) as noted elsewhere [1]. The quantity $\hat{R}=1-\frac{\frac{1}{N}\sum_{i=1}^{N} \hat{\sigma}_{\hat{\theta}_{i}}^{2}}{\hat{\sigma}_{\hat{\theta}}^{2}}$, in which $\hat{\sigma}_{\hat{\theta}}^{2}$is the sample variance of the $\hat{\theta}$ and N is the number of subjects, can then serve as an estimator for the reliability of $\hat{\theta}$as an estimate of$\theta$. Similarly, in Bayesian MCMC estimation, values of $\hat{\sigma}_{\hat{\theta}_{i}}^{2}$ can be obtained directly from the posterior distribution of $\hat{\theta}_{i}$ and then $\hat{R}$is again obtained as $\hat{R}=1-\frac{\frac{1}{N}\sum_{i=1}^{N} \hat{\sigma}_{\hat{\theta}_{i}}^{2}}{\hat{\sigma}_{\hat{\theta}}^{2}}$.

**An Alternative from the field of psychometrics.**

An alternative for cases in which MLE or Bayesian MCMC approaches are not used to estimate $\theta$ (e.g., [2]) or when one wishes for a more semi-parametric method of estimating reliability, we offer an approach adapted from the field of psychometrics. By more semi-parametric we mean a method that depends only on the most minimal distributional assumptions about the variables under study (i.e., that all have finite first and second moments) and only on the most minimal assumptions about the genetic model (specified below).

Voight et al. [3] provided an interesting graphic analysis presaging this approach. They used Structure to estimate individual ancestry values for a sample of Mexican Americans using only the odd-numbered chromosomes and then repeated the estimation using only the even-numbered chromosomes. Visual inspection of the two sets of results showed a marked similarity, but no formal analysis was conducted. The extension of this approach to a formal reliability assessment procedure is straightforward. First, it is important to note that by the law of independent assortment, conditional on individual ancestry and the product of parental ancestry, what one inherits on one chromosome is independent of what one inherits at any other chromosome. Thinking of the chromosome-specific estimates $\hat{\theta}_{ij}$ (j denotes the j^th^ chromosome) as manifest variables [4], or ‘test items’ that are markers for the latent variable $\theta_{i}$, the law of independent assortment implies that the chromosome-specific measurement errors (i.e., $\hat{\theta}_{ij}-\theta_{i}$) are independent for all j. Given this recognition, one could correlate the ancestry estimates obtained from the odd chromosomes in a dataset with the corresponding estimates obtained using only the even chromosomes and use that correlation as a reliability estimate. Psychometricians refer to this as ‘split-half reliability’ estimation. This will give the reliability of estimates that use only half of the available data. However, the well-known Spearman-Brown prophecy formula [5,6] was developed to correct for this type of underestimation. Of course, one might ask if there are other ways to split the data than by odd- and even-numbered chromosomes and this question leads to Cronbach’s coefficient α [7], which is the average of all possible corrected split-half reliability coefficients calculated using W items. In this context, W represents the number of chromosomes on which admixture is estimated and Cronbach’s α is $\frac{W}{W-1}\left(1-\frac{\sum_{j=1}^{W} S_{\hat{\theta}_{j}}^{2}}{S_{\hat{\theta}}^{2}}\right)$, in which $S_{\hat{\theta}_{j}}^{2}$is the sample variance of the chromosome-specific estimates of $\theta$ for the j^th^ chromosome and $S_{\hat{\theta}}^{2}$ is the sample variance of the individual estimates of $\theta$ obtained using all chromosomes. Given that the chromosomes will not all be equally informative due to different lengths and numbers of markers, the formula will need to be adjusted to take into account the fact that the chromosome-specific admixture scores will be given different weights in estimating individual ancestry. Cronbach’s α will allow investigators to estimate the reliability of ancestry-related variable estimates without reference to external information or assumptions about the genetic model except that there is no segregation distortion and that transmission probabilities are independent for loci on different chromosomes. If one is dealing with a random sample of gametes, these two assumptions imply that $E\left(\hat{\theta}_{ij}\right)=\theta_{i}$ and that the differences $\hat{\theta}_{ij}-\theta_{i}$ are independent for all j. Although this may not be strictly true in non-random samples of gametes, we expect that any deviations will be of minor consequence.

**A second alternative for use with family data**.

MacLean and Workman [5,6] offered a simple observation that allows one to estimate the reliability of individual admixture estimates used as estimates of individual ancestry. By definition, full siblings have identical values of individual ancestry variables. Therefore, if one has a sample of full sibling pairs, one can calculate the sibling correlation of the $\hat{\theta}$ as a direct measure of reliability. This implies that in a study of siblings, the best estimate of each individual’s $\theta$ is not the value obtained by examining only their marker data. Rather, the best estimate for each person is the simple arithmetic mean (or inverse-variance-weighted if one wanted to allow for differential reliability within sibship) of the estimates initially obtained for an individual and each of their siblings separately. As with other methods, in a sibling pair study the reliability estimate obtained by utilizing the sibling correlation would need to be corrected for the number of siblings. With sibling pairs, one can use Cronbach’s α formula now letting W denote the number of siblings (2 for sibpairs), $S_{\hat{\theta}_{j}}^{2}$ the sibling-specific variances, and $S_{\hat{\theta}}^{2}$ the sample variance of the within-sibship mean estimates of ancestry. Conceivably, one should be able to extend this approach to general pedigrees, though this requires more assumptions (e.g., that the parents of subjects in the analysis did not mate assortatively for individual ancestry).

**ESTIMATING INDIVIDUAL ANCESTRY AND THE PRODUCT OF PARENTAL ANCESTRIES.**

As we emphasized above, the need for estimating aspects of parental ancestries does not require that parents be included in the studies. Let $\Theta$denote the vector of ancestry-related values we wish to estimate for an individual, $\theta_{1}$ their individual ancestry (A) from population V, $\theta_{2}$ the product of their parental ancestries (P_1i_P_2i_), X the observed data, k the number of markers studied, I(·) the indicator function, and the number of alleles inherited from population V at the j^th^ locus. If all loci are unlinked and are located on autosomes, the likelihood of $\Theta$given the data is

$L\left(\Theta\left|X\right.\right)=\prod_{j=1}^{J} \left[I\left(v_{j}=0\right)\left(1-2\theta_{1}+\theta_{2}\right)+I\left(v_{j}=1\right)2\left(\theta_{1}-\theta_{2}\right)+I\left(v_{j}=2\right)\theta_{2}\right]$. This likelihood for ancestry and the product of parental ancestries can also be expressed by the equation:

$L=\mathop{\Pi}\limits_{j=1}^{J} (1-2\theta_{1}+\theta_{2})^{x_{j0}}(2(\theta_{1}-\theta_{2}))^{x_{j1}}\theta_{2}^{x_{j2}}$, where J is the number of loci used to estimate ancestry, θ_1_ = (P_1_+P_2_)/2, and θ_2_ = P_1_*P_2_. It is noted that only three combinations of x_j0_, x_j1_, and x_j2_ are possible. These combinations are a) x_j0_ = 1, x_j1_ = 0, and x_j2_ = 0 if and only if 0 alleles from population V have been inherited at locus k, b) x_j0_ = 0, x_j1_ = 1, and x_j2_ = 0 if and only if 1 allele from population V has been inherited at locus k, and c) x_j0_ = 0, x_j1_ = 0, and x_j2_ = 1 if and only if 2 alleles from population V have been inherited at locus j. The maximum likelihood estimator (MLE) of θ_2_ in this fully informative case is $\hat{\theta}_{2}=\frac{\sum_{j=1}^{J} X_{j2}}{J}$.

However, x_j0_, x_j1_, and x_j2_ can be re-expressed using 2 indicator variables representing ancestry, one for each allele at the j^th^ locus. Let the variable A_j1_ represent the event that allele 1 at the j^th^ locus came from population V. Let A_j1_ = 1 when the first allele at the j^th^ locus came from population V and let A_j1_ = 0 otherwise. Let I(A_j1_ = 1) be an indicator variable for the event A_j1_ = 1. Define A_j2_ and I(A_j2_= 1) similarly for allele 2 at the j^th^ locus. Using these indicator variables, x_j0_ can be expressed as (1- I(A_j1_ = 1))* (1- I(A_j2_ = 1)), x_j1_ can be expressed as (1- I(A_j1_ = 1))*I(A_j2_ = 1)+ (1- I(A_j2_ = 1))*I(A_j1_ = 1)), and x_j2_ can be expressed as I(A_j1_ = 1)*I(A_j2_ = 1). Using substitution, the MLE of θ_2_ is $\hat{\theta}_{2}=\frac{\sum_{j=1}^{J} I(A_{j1}=1)*I(A_{j2}=1)}{J}$. By the invariance properties of MLEs [8], maximum likelihood estimates of θ_2_ in the less than fully informative case can be calculated by substituting MLEs of I(A_j1_ = 1) and I(A_j2_ = 1) into the formula for $\hat{\theta}_{2}$. The EM algorithm approach presented in Tang et al. [9] provides MLE estimates for I(A_j1_ = 1) and I(A_j2_ = 1) for all ancestry informative loci. Hence, the estimation of θ_2_ or P_1_*P_2_ for individuals is straightforward. It should be noted that the MLE of θ_1_ under our notation provides the same functional form of the MLE for ancestry that Tang et al. [9] provides.

After convergence of the algorithm, the method has estimated the probability that the l^th^ allele ( l = 1, 2) at the j^th^ marker arose from population k. A researcher need only multiply the 2 probabilities available at the each of the j markers, sum across the j markers, and divide the sum by j to obtain the estimate of P_1i_*P_2i_ for the any individual. In equation form, the estimate of the product of parental ancestries for the i^th^ individual is :

$est(p_{1i}*p_{2i})=\frac{\sum_{j=1}^{J} p_{_{1ij}}(k)*_{p_{2ij}}(k)}{J}$. We have modified our programming of Tang et al.’s [9] algorithm to provide these estimates and while accurate estimation of individual ancestry and the product of parental ancestries do require marker information from non-admixed representatives of the original populations, the procedure does not require marker information for an individual’s parents.

Use of the above likelihood formula requires that the $v_{j}$are known. Most investigators are satisfied to use such an expression even when markers may be linked (e.g., [9,1,10,11]). Tang et al. ([9], p. 291) offer a justification for this and present evidence that it does not have a serious adverse impact on the estimates in at least some circumstances. However, it is clear that methods of producing confidence intervals that assume unlinked markers can produce intervals that are too narrow if markers are in fact linked, implying that such methods will overestimate measurement reliability in such cases ([9]; p. 298-299; [12]). In contrast, we note that the semi-parametric methods of estimating reliability offered above based on Cronbach’s α or sibling correlations do not suffer from this limitation. Extensions to linked markers are available (e.g., [13], Equations 2 and 3; [14], Equation 1). Also available are extensions to situations in which markers are not perfectly informative [1], ancestral allele frequencies are not known without error [9] and markers are located on the sex chromosomes [15]. Additionally, it should be acknowledged that ancestral allele frequencies and even the number of contributing ancestral populations are usually in doubt [16]. As was recently noted by the Race, Ethnicity, and Genetics Working Group ([17]; pp. 551): “In many parts of the world, groups have mixed in such a way that many individuals have relatively recent ancestors from widely separated regions. Although genetic analyses of large numbers of loci can produce estimates of the percentage of a person’s ancestors coming from various continental populations…these estimates may assume a false distinctiveness of the parental populations, since human groups have exchanged mates from local to continental scales throughout history.” In such cases, the approach of Zhao and colleagues [2,18,19], which uses principal components analysis to extract vectors accounting for background genetic covariation among variables representing dummy coded genotypes (as opposed to just allele counts, allowing for analogues of both our $\theta_{1}$ and $\theta_{2}$ to be estimated) at marker loci throughout the genome, should prove useful.

**References**

1. Pfaff CL, Barnholtz-Sloan J, Wagner JK, Long JC (2004) Information on ancestry from genetic markers. Genetic Epidemiology 26: 305-315.

2. Zhang SL, Zhu XF, Zhao HY (2003) On a semiparametric test to detect associations between quantitative traits and candidate genes using unrelated individuals. Genet Epidemiol 24: 44–56.

3. Voight BF, Cox NJ, Pritchard JK (2002) Detection of population structure in Starr County Mexican-Americans. American Journal of Human Genetics 71: 352.

4. Loehlin J (1987) Latent variable models. Hillsdale: Lawrence Erlbaum Associates.

5. Maclean C, Workman P (1973) Genetic studies on hybrid populations .2. Estimation of distribution of ancestry. Annals of Human Genetics 36: 459-465.

6. Maclean C, Workman P (1973) Genetic studies on hybrid populations .1. Individual estimates of ancestry and their relation to quantitative traits. Annals of Human Genetics 36: 341-351.

7. Cronbach L (1951) Coefficient alpha and the internal structure of tests. Psychometrika 16: 297-334.

8. Cassella, Burger (1990) Statistical Inference. Belmot, CA: Duxbury Press. 294 p.

9. Tang H, Peng J, Wang P, Risch NJ (2005) Estimation of individual admixture: Analytical and study design considerations. Genet Epidemiol 28: 289–301.

10. Barnholtz-Sloan JS, Chakraborty R, Sellers TA, Schwartz AG (2005) Examining population stratification via individual ancestry estimates versus self-reported race. Cancer Epidemiology Biomarkers & Prevention 14: 1545-1551.

11. Tsai H, Choudhry S, Naqvi M, Rodriguez-Cintron W, Bruchard EG, Ziv E (2005) Comparison of three methods to estimate genetic ancestry and control for stratification in genetic association studies. Human Genetics. 118:424-433.

12. Excoffier L, Estoup A, Cornuet JM (2005) Bayesian analysis of an admixture model with mutations and arbitrarily linked markers. Genetics 169: 1727-1738.

13. Falush D, Stephens M, Pritchard JK (2003) Inference of population structure using multilocus genotype data: Linked loci and correlated allele frequencies. Genetics 164: 1567–1587.

14. Zhang C, Chen K, Seldin MF, Li HZ (2004) A hidden Markov modeling approach for admixture mapping based on case-control data. Genet Epidemiol 27: 225–239.

15. Patterson N, Hattangadi N, Lane B, Lohmueller KE, Hafler DA, et al. (2004) Methods for high-density admixture mapping of disease genes. Am J Hum Genet 74: 979–1000.

16. Salas A, Carracedo A, Richards M, Macaulay V (2005) Charting the ancestry of African Americans. American Journal of Human Genetics 77: 676-680.

17. Race Ethnicity and Genetics Working Group (2005) The Use of Racial, Ethnic and Ancestral Categories in Human Genetics Research. American Journal of Human Genetics 77: 519-532.

18. Chen HS, Zhu X, Zhao H, Zhang S (2003) Qualitative semi-parametric test for genetic associations in case-control designs under structured populations. Ann Hum Genet 67: 250–264.

19. Zhu XF, Zhang SL, Zhao HY, Cooper RS (2002) Association mapping, using a mixture model for complex traits. Genetic Epidemiology 23: 181-196.
